# Antifungal activity of *Carica papaya* fruit extract against *Microsporum canis*: *in vitro* and *in vivo* study

**DOI:** 10.3389/fmicb.2024.1399671

**Published:** 2024-05-13

**Authors:** Salma Aljuhani, Humaira Rizwana, Abeer S. Aloufi, Saad Alkahtani, Gadah Albasher, Hadeel Almasoud, Rasha Elsayim

**Affiliations:** ^1^Department of Botany and Microbiology, College of Science, King Saud University, Riyadh, Saudi Arabia; ^2^Department of Biology, College of Science, Princess Nourah bint Abdulrahman University, Riyadh, Saudi Arabia; ^3^Department of Zoology, College of Science, King Saud University, Riyadh, Saudi Arabia

**Keywords:** *Microsporum canis*, *Carica papaya*, dermatophytes, antifungal, tinea capitis

## Abstract

**Background:**

T*inea capitis* (*T. capitis*), commonly known as scalp ringworm, is a fungal infection affecting the scalp and hair. Among the causative agents, *Microsporum canis* (*M. canis*) stands out, often transmitted from cats to humans (*zoonotic disease*). In this study, we investigated the efficacy of *Carica papaya* (*C. papaya*), fruit extract against dermatophytes, particularly *M. canis*, both *in vitro* and *in vivo*. Additionally, we aimed to identify the active compounds responsible for suppressing fungal growth and assess the toxicity of *C. papaya* on human cells.

**Methodology:**

It conducted in two parts. First, *In Vitro* Study include the preparation of *C. papaya* fruit extract using methanol as the solvent, Phytochemical analysis of the plant extract including Gas chromatography–mass spectrometry (GC–MS) and Fourier-transform infrared spectroscopy (FTIR) was conducted, Cytotoxicity assays were performed using HUH-7 cells, employing the MTT assay (1-(3-(4,5-dimethylthiazol-2-yl)-2,5-diphenyl tetrazolium bromide), Antimicrobial activity against *M. canis* was evaluated, including: Zone of inhibition (ZI), Minimum inhibitory concentration (MIC), Minimum fungicidal concentration (MFC), *M. canis* cell alterations were observed using scanning electron microscopy (SEM) and transmission electron microscopy (TEM). Second, *In Vivo*, Albino Wistar male rats were included.

**Results:**

The phytochemical analysis of the methanolic extract from papaya revealed several functional groups, including hydroxyl, ammonia, alkane, carbonate, and alcohol. Additionally, the GC–MS analysis identified 15 compounds, with xanthosine and decanoic acid being the predominant components. The methanolic extract of papaya fruits demonstrated potent antifungal activity: ZI = 37 mm, MIC = 1,000 μg/mL, MFC = 1900 μg/mL, MTT results indicated lower cytotoxicity of the fruit extract at concentrations of 20 μg/mL, 50 μg/mL, 100 μg/mL, 150 μg/mL, and 200 μg/mL, The IC50 revealed a significant decrease in cell viability with increasing extract concentration. Notably, papaya extract induced considerable alterations in the morphology of *M. canis* hyphae and spores. In animal tissue, improvements were observed among the group of rats which treated with Papaya extract. This study highlights the potential of *C. papaya* fruits as a natural antifungal agent, warranting further exploration for clinical applications.

## Introduction

*Microsporum canis* (*M. canis*), is a type of fungus that can cause tinea capitis, a disorder characterized by severe itching of the scalp, red scaly papules around hair shafts, and hair loss in both humans and animal. This organism is found worldwide (Brillowska-Dabrowska et al., 2013; Mao et al., 2014).

*Microsporum canis* is a also cause invasive infections in immunocompromised patients (Mao et al., 2014). It is highly infectious and can be zoonotic through direct physical contact or indirect contact with fungus-contaminated materials (Yu et al., 2004; Mao et al., 2014). *M. canis* is one of the most common dermatophytes to cause human tinea capitis in Europe and South America (Fernandes et al., 2001; del Boz et al., 2011). The prevalence of dermatophyte infections worldwide is influenced by several factors, involving climate, gender, age, lifestyle, human migration, cultural habits, and socioeconomic status (Alshehri et al., 2021). A limited study of the prevalence of *M. canis* in Saudi Arabia was conducted in 2021, which found that among the 10,021 samples analyzed, 3,040 (30.33%) were positive for fungi and only 398 (3.97%) were dermatophytes. *Microsporum species* was the most common dermatophyte accounting for 50.5% (n = 201) (Alshehri et al., 2021).

There are many oral and topical antifungal protocols available for treating *M. canis* infection. The most used drugs are azoles, polyenes, allylamines, and griseofulvin (Aneke et al., 2018). But the efficacy of these drugs and treatment protocols is variable, with treatment failure more than 40% of patients possibly due to resistance phenomena (Aneke et al., 2018). It is necessary to find an eco-friendly drug with low side effects and toxicity due to the urgency of these phenomena. Medicinal plants are the most used antimicrobial agents for treating fungi and have been used traditionally. Papaya (*Carica papaya Linn*), a fruit that belongs to the Caricaceae family, is recognized worldwide for its nutritional and beneficial properties. The various parts of the papaya plant have been used for medicinal uses since ancient times (Singh et al., 2020). The ripe fruit is used to treatment chronic forms of skin indurations, sinuses, Chronic skin ulcers, bleeding piles and dyspepsia in India, stomachic, digestive, diuretic, sedative and tonic (Singh et al., 2020). Recently, considerable literature has grown up around the theme of papaya’s medicinal qualities, and they found that Papaya is presented strong medicinal properties including antibacterial, antiviral, antitumor, hypoglycemic, and anti-inflammatory properties.

Although active components are extracted from all parts of the papaya plant, the concentration of these components alters from structure to structure. For medicinal purposes, parts known to contain the highest concentration of the principles are preferred (Aruljothi et al., 2014). These parts include the leaves, stem, barks, roots, bulks, corms, rhizomes, woods, flowers, fruits, and seeds. Chymopapain and papain are two important bioactive compounds present in *C. papaya*. Papaya leaves are used to for the treatment of malaria. Papaya’s leaves contain karpain, a compound that kills microorganisms. Additionally, Papaya’s leaf contains phenolic compounds such as protocatechuic acid, p-coumaric acid, 5, 7-dimethoxycoumarin, caffeic acid, kaempferol, quercetin, and chlorogenic acid (Aruljothi et al., 2014).

There is a growing body of literature that recognizes the role of *C. papaya* in antimicrobial treatment. One of these studies was conducted in India which tested the leaf extract of *C. papaya*, against wound infection causing bacteria such as: *Klebsiella pneumoniae*, *Pseudomonas aeruginosa*. *Escherichia coli*, *Proteus vulgaris*, and *Staphylococcus aureus*. They found that Papaya’s extract pronounced antibacterial effect on gram negative bacteria especially *Pseudomonas* sp. (Aruljothi et al., 2014). According to many studies, papaya’s fruits are a potent source of antibacterial agents that can be used by pharmaceutical industries to produce medications (Hariono et al., 2021; Sharma et al., 2022). Additionally, researchers in China found that papaya seed essential oil (EO) has promising anticandidal activity and identified *C. papaya* as a potential natural source of antifungal agents (He et al., 2017). No previous study has been conducted to investigate the antifungal activity of *C. papaya* fruits in Saudi Arabia or anywhere else in the world. The objective of this study was to evaluate the effectiveness of *C. papaya* fruits in overcoming dermatophytes, particularly *M. canis*, and to identify the active compounds that suppress fungal infection and the toxicity’s degree of *C. papaya* on the human cell.

## Methodology

### Collection of the plant

Papaya fruits were purchased from a local market, rinsed with tap water, sectioned into bite-sized pieces, and then left to dry in a shaded area.

### Preparation of extracts

*Carica papaya* fruits was dried and grinded to extract with methanol. The plant’s fruit powder (100 grams) was individually soaked in 500 mL of solvent and placed on the orbital shaker (Benchmark BT3001 Orbi-Shaker, Marshall scientific, Virginia, USA) for a duration of 72 h (Harborne, 1998; Nigussie et al., 2021). The extracts were filtered using Whatman No1 filter paper. Methanol was then removed from the extracts using normal evaporation in a sterile laminar cabinet for 5 days (Al-Saiym et al., 2015). After complete drying, the extracts were stored at room temperature until used for further study.

### Phytochemical screening of the extracts

The active compounds present in *C. papaya* extract, were detected by conducting a phytochemical analysis. The compounds present in the Papaya’s fruit extract were analyzed using a gas chromatography (GC–MS) device (Agilent 7890A) coupled with a mass spectrometer (5975C, Agilent Technologies, Santa Clara, CA, USA). The GC–MS system included a DB-5MS column (30 m length, 0.25 mm inner diameter, 0.25 μm film thickness), a Triple-Axis detector, and a liquid sampler. Prior to analysis, a 22 μm membrane filter was used to purify 1 mL of the extract. The extract was then introduced into the system as a 1 L aliquot. The injection and column temperatures were set at 280°C and 300°C, respectively. Helium served as the mobile phase with a flow rate of 1 mL/min. The electron ionization energy was set at 70 eV. To identify the different functional groups, present in the methanolic plant extract, we used FTIR in the range of 400–4,000 cm^−1^ (Parkin Elmer, Spectrum BX, Waltham, UK). were used to study the functional groups and compounds that make the extract potent to use as an antimicrobial agent.

### Cytotoxicity assays 1-(3-(4, 5-dimethyl thiazol-2-yl)-2, 5-diphenyl tetrazolium bromide) MTT assay

In order to investigate Papaya’s cytotoxicity, we did the MTT assay which is a colorimetric assay used to determine the number of viable cells in culture (Cree, 2011). It relies on the intracellular reduction of MTT to formazan by mitochondrial dehydrogenases.

The MTT powder was dispersed in culture medium placed with 20,000 of HUH-7 cell lines at 0.5 mg/mL. After exposure to the methanolic papaya extract for 48 h, the culture medium was removed from the 96-wells plate. Then, cells were washed twice with 0.05 M Phosphate-buffer saline (PBS), MTT solution was added (100 μL/well), and incubated for 3 h at 37°C. After incubation, cells were washed with PBS, and Dimethyl sulfoxide (DMSO) 10%, was added to dissolve formazan crystals (100 μL/well). Finally, plates were incubated on a shaker for 15 min at room temperature to ensure complete solubilization. Absorbance was measured to each sample at 570 nm using a microplate reader (BioTek Synergy H1, Turku, Finland). The effect of the Ramucirumab on cells was measured by counting viable cells by calculating the concentration that inhibits 50% of cell line growth (IC50) determined by the dose–response curve using a software program (Origen Pro 8.5) after repeating the experiment three times (Monks et al., 1991).

### Microbial susceptibility testing of *Carica papaya* extracts

#### Preparation of tested fungi

Dermatophytes (*M. canis* ATCC-36299) was used in antifungal essays were obtained from the collection of Mycology Laboratory (LM), Microbiology department, King Khalid hospital, Riyadh, Saudi Arabia. *M. canis* was maintained in sabouraud dextrose agar (SDA) at 28°C for 10 days and stored at 4°C until used.

### Inoculum preparation

Stock inoculum suspension of *M. canis* was prepared from 10-day culture in SDA at 28°C to induce sporulation. Fungal colonies were covered with 5 mL of sterile saline solution (NaCl 0.85% w/v), by sterile loop the SDA’s surface gently scraped to obtain mixture of fungal units, which was transferred to a sterile tube of distilled water. The turbidity of the final inoculum was standardized according to McFarland scale 0.5 tube and adjusted for presenting the fungal population of 10 (Alshehri et al., 2021) colony former units (CFU) (Alastruey-Izquierdo et al., 2015).

### Antifungal activity screening

As a preliminary test for evaluating the potential activity of the extracts and the tested antibiotic Itraconazole 10 mg/mL (Livealth Biopharma private Ltd. Mahrashtra, India), as control (Aneke et al., 2021). The well diffusion method was used for antifungal activity screening. 200 μL of each extract (7.5 mg/mL), was added to each well. All plates were incubated for 10–14 days at 28°C. The diameter of the fungal growth inhibition zone was measured and expressed in millimeters at the end of the incubation period. Antifungal activity was considered positive when the geometric mean values of growth inhibition zone in two independent essays were equal to or greater than 10 mm diameter (Alastruey-Izquierdo et al., 2015; Aneke et al., 2021; Rasha et al., 2021).

### Determination of minimum inhibitory concentration (MIC)

The MIC values were determined for *M. canis* after we observed a promised results with methanol extract of *C. papaya* only. Broth macro dilution bioassay was used to determine the MIC (Alastruey-Izquierdo et al., 2015). The tubes were prepared by dispensing 1 mL of doubled concentrated Sabouraud dextrose broth (SDB) into each tube. From the stock emulsion of each tested extract (30 mg/mL), 1 mL was added to the first tube, resulting in a concentration of 15 mg/mL. Then, 1 mL from their serial dilutions were transferred into the rest of tubes, excluding the last ones. The last tube contained 1 mL of broth inoculated with fungal inoculum to confirm the cell viability (viability control). At the same way positive control was carried out with standard antifungal using Itraconazole 10 mg\mL. In the tested tubes containing SDB and the different concentration of the tested extracts, 1 mL of *M. canis* were matched 0.5 McFarland unite (1 to 2 × 10^8^ CFU/mL) which was prepared, then diluted 1:150, resulting in a tube containing approximately 1 × 10^6^ CFU/mL. This mixture results to a 1:2 dilutions of each antimicrobial concentration, and a 1:2 dilution of the inoculum. The subsequent 1:2 dilution of inoculum brought the final inoculum to 5 × 10^5^ CFU/mL. Following this, the samples were incubated overnight in shaker incubator (180 rpm) at 28°C for10 days. The MIC values were determined by visual inspection of the growth inhibition of each well compared with that of the control (without drugs) well (Alastruey-Izquierdo et al., 2015; Nigussie et al., 2021).

### Determination of the minimum fungicide concentration MFC

The MFC was determined the macro dilution method to verify if the inhibition was reversible or permanent (Alastruey-Izquierdo et al., 2015). Aliquot of 20 μL from the wells that did not show growth in MIC procedure was transferred to SDA plates. The plates were incubated at 28°C and being read 10 days of incubation. MFC was defined as the lowest concentration in which no visible growth occurred when subculture on the plates contained broth without antifungal products.

### SEM and TEM analysis of *Carica papaya*-treated *Microsporum canis*

The microstructural changes in the *M. canis* cells treated with *C. papaya* were observed by a scanning electron microscopy (SEM) device (JEOL model, JSM761OF, Tokyo, Japan). A total of 1 mL of all tested organism suspensions (10^8^ CFU/mL) was mixed with 1 mL of each tested compound, which resulted in a mixture with a 0.5 mg/mL concentration. The mixture was then incubated overnight at 28°C. In a salt-free Lysogeny broth (LB) medium, two controls were prepared: a positive control consisting of an organism treated with Itraconazole. After the incubation, the samples were washed with saline solution and then centrifuged at 1500× g. The samples were fixed with 2.5% glutaraldehyde (4°C, 2 h), followed by washing with phosphate buffer (pH 7.20). The samples were again fixed in 1% osmium tetroxide, dehydrated using an ascending ethanol series, and then subjected to critical point drying. Finally, the samples were coated with Au–Pd (80:20) using a Polaron E5000 sputter coater, and they were observed on a scanning electron microscope equipped with an SE detector working at 25 kV. The TEM samples were prepared using identical buffers and dehydration protocols as those used for the SEM samples. This was followed by dehydration process using an acetone series and then embedding in epoxy resin. Sections of extreme thinness were sliced and placed on grids coated with formvar. These sections were then stained with a 3% solution of uranyl acetate. Observations of the samples by TEM were applied by a JEM-2100F microscope from JEOL Ltd., located in Peabody, MA, USA (Ramadan et al., 2009; CLSI, 2012; Venkatachalam et al., 2015). And by SEM using Polaron E5000 sputter coater, Quorum Technologies, Laughton, UK.

### The effects of papaya fruit extract on *Microsporum canis in vivo*

Albino Wistar male rats, aged between 6–8 weeks, were obtained from the Animal House at the College of Pharmacy in King Saud University, Riyadh, Saudi Arabia. These rats were individually accommodated in plastic cages filled with wood shavings. They were subjected to a consistent 12-h light–dark cycle and provided with standard laboratory rat food and purified tap water (Liu et al., 2017; Rasha et al., 2021). The Animal care and use committee at King Saud University granted approval for all animal experiments (Ethics Reference Number: KSU-SE-1978). The anesthesia process for the rats followed to the guidelines set forth by the office of Research Institutional Animal Care and Use at the University of California, San Francisc (Sharma et al., 2019). The combination of Ketamine and xylazine combination was administered as an intraperitoneal injection. The dosage used was 0.5 mL per 100 g of body weight, which equates to 100 mg of ketamine and 5 mg of xylazine for each kilogram of body weight (Sharma et al., 2019). After anesthetized the experimental rats, we removed their dorsal hair using a shaver, followed by washing with a lather and water. We scratched the shaved area to cause superficial wounds to enhance the dermatophyte’s infection. The total number of rats was 45, which were divided into three equal groups: group 1 (control) which infected and left without any treatment, group 2 which infected and treated with Canesten ointment (0.2 gm clotrimazole) and group 3 infected and treated with papaya extract ointment (10 mg/mL). After six days of the inducing the infection, we took swab samples from each wound to confirm that those wounds had been infected with *M. canis* and we collected tissue samples on this day and on day 9 and 15 post the infection.

The process of creating gel-based ointments was adapted from the Elsayim method with minor adjustments. The formulation involved combining equal volumes of Polyethylene Glycol (PEG) 400 and 2000, along with papaya fruit extract at a concentration of 10 mg/mL. The mixtures were then heated to a temperature of 65°C for a duration of 5 min (Tiwari et al., 2018; Rasha et al., 2021).

The process of healing skin infections and re-epithelialization was studied using Hematoxylin and Eosin (H&E) staining on tissue samples obtained from the infected skin areas. This was followed by evaluations of their structure and tissue characteristics (Yoshioka et al., 2018; Zhang et al., 2019). These tissue slices were examined under a light microscope (Nikon, Eclipse i80. Tokyo, Japan), and photographs were captured at various zoom levels with a digital camera attached to the microscope (OXM 1200C, Nikon, Japan).

The data obtained from MTT and *in vivo* studies were analyzed by using ANOVA with SPSS statistical software version 22 (SPSS Inc., Chicago, IL, USA).

## Results

**Figure 1:**
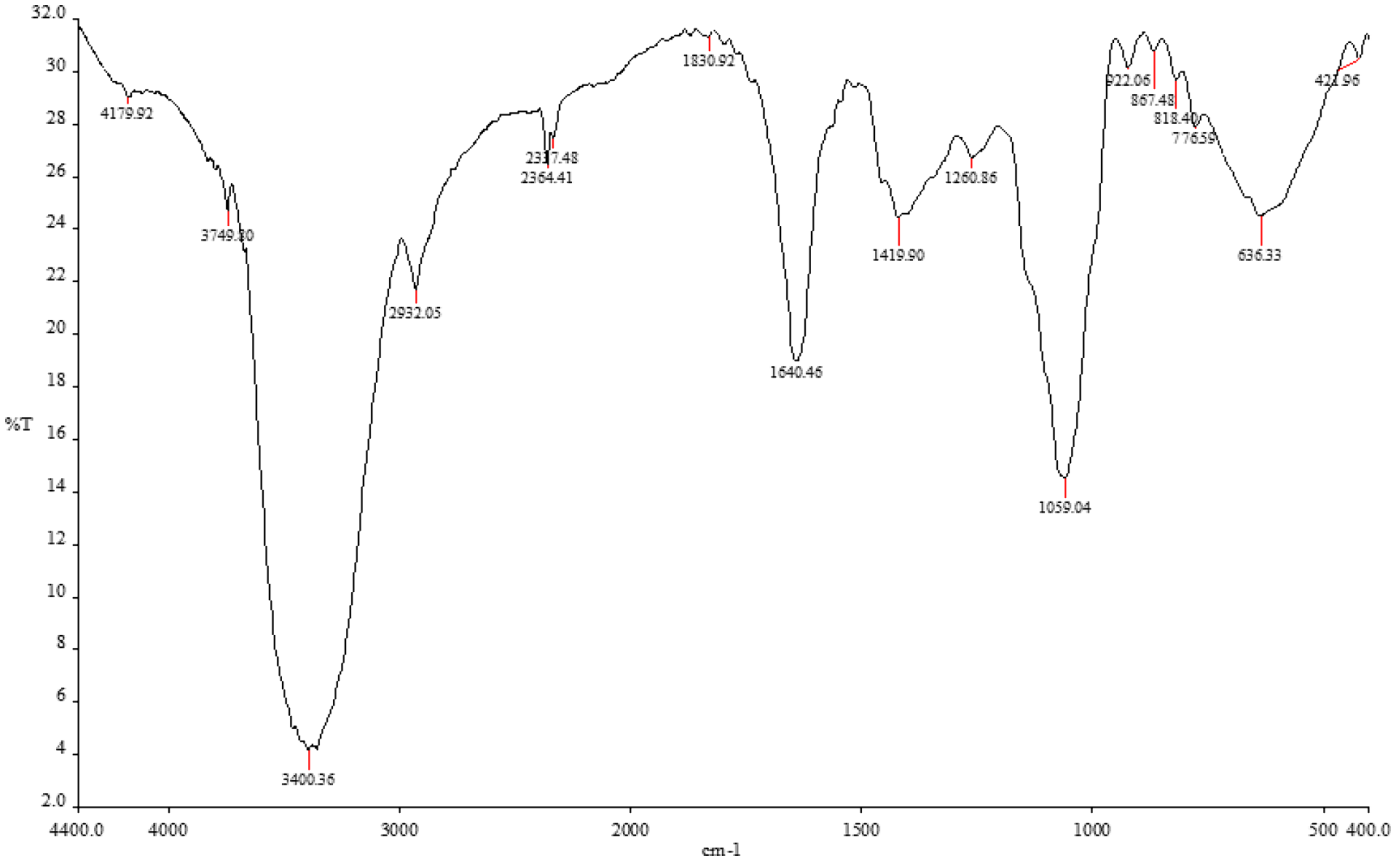
The functional group of *Carica papaya* extracted by methanol by FTIR analysis.

Given the high prevalence of tinea capitis in Saudi Arabia compared to other dermatophytes infection, we sought to develop a new, eco-friendly, and effective treatment to combat the causative agent of this condition, *M. canis*. We selected the fruit of the papaya plant for its potential to inhibit this fungus, extracting its active components that could play a role in eradicating the fungus (Singh et al., 2020). Our investigates began with the methanol extraction of papaya fruits, followed by testing the functional group identified in the extract by FTIR analysis (as shown in Figure 1; Table 1).

**Table 1:** FTIR analysis of *Carica papaya* extracted by methanol.

Absorption (cm^−1^)	Peak details (Appearance)	Group	Compound class	Reference standard (cm^−1^)
1. 4179.92		Alcohol	O-H stretch	>3,500
2. 3749.80	Strong, broad	Alcohol	O-H stretch	>3,500
3. 3400.36	Medium	Aliphatic primary amine	N-H stretching	3,400–3,500
4. 2932.05	Medium	Alkane	C-H stretching	2,840–3,000
5. 2364.61				
6. 2337.48		Carbonate	O=C=O stretch	2,275–2,349
7. 1830.92	Weak	Aromatic compound	C-H bending	1,650–2000
8. 1640.46	Medium	Alkene	C=C stretching	1,638–1,648
	Strong	Alkene	C=C stretching	1,638–1,648
9. 1419.90	Medium	Carboxylic acid	O-H bending	1,395–1,440
10. 1260.86		Alkyl aryl ether	C-O stretch	1,200–1,275
11. 1059.04		Primary alcohol	C-O stretching	1,050–1,085
12. 922.06		Alkene	C=C bend	
13. 867.48		1,2,4-trisubstituted 1,3-disubstituted	C-H bending	860–900
14. 818.40		1,4-disubstituted 1,2,3,4-tetrasubstituted	C-H bending	790–830
15. 776.59		1,2,3-trisubstituted	C-H bending	760–800

The methanolic extract of papaya showed a broad, strong peak at 4179.92 cm^−1^ and 3749.80 cm^−1^, representing the O–H stretching vibration of the Alcohol. This peak was blue-shifted with decreased intensity to 3,400 cm^−1^ were related to the N–H stretching vibrations of aliphatic primary amin. The extract also showed a medium absorption peak at 2932.05 cm^−1^, 1830.92 cm^−1^ and 867.48 cm^−1^, which correlated to asymmetrical stretching vibrations of Alkane group and 1,2,3-trisubstiuted compounds. The strong bands at 1640.46 cm^−1^ observed in the papaya extract spectrum were ascribed to the C=C of Alkene. The band at 2337 cm^−1^ was related to carbonate. Peaks at 1260 cm^−1^ and 1,059 cm^−1^ corresponded to C-O stretching vibrations of alkyl aryl ether and primary alcohol, respectively.

### GC-mass analysis

**Figure 2:**
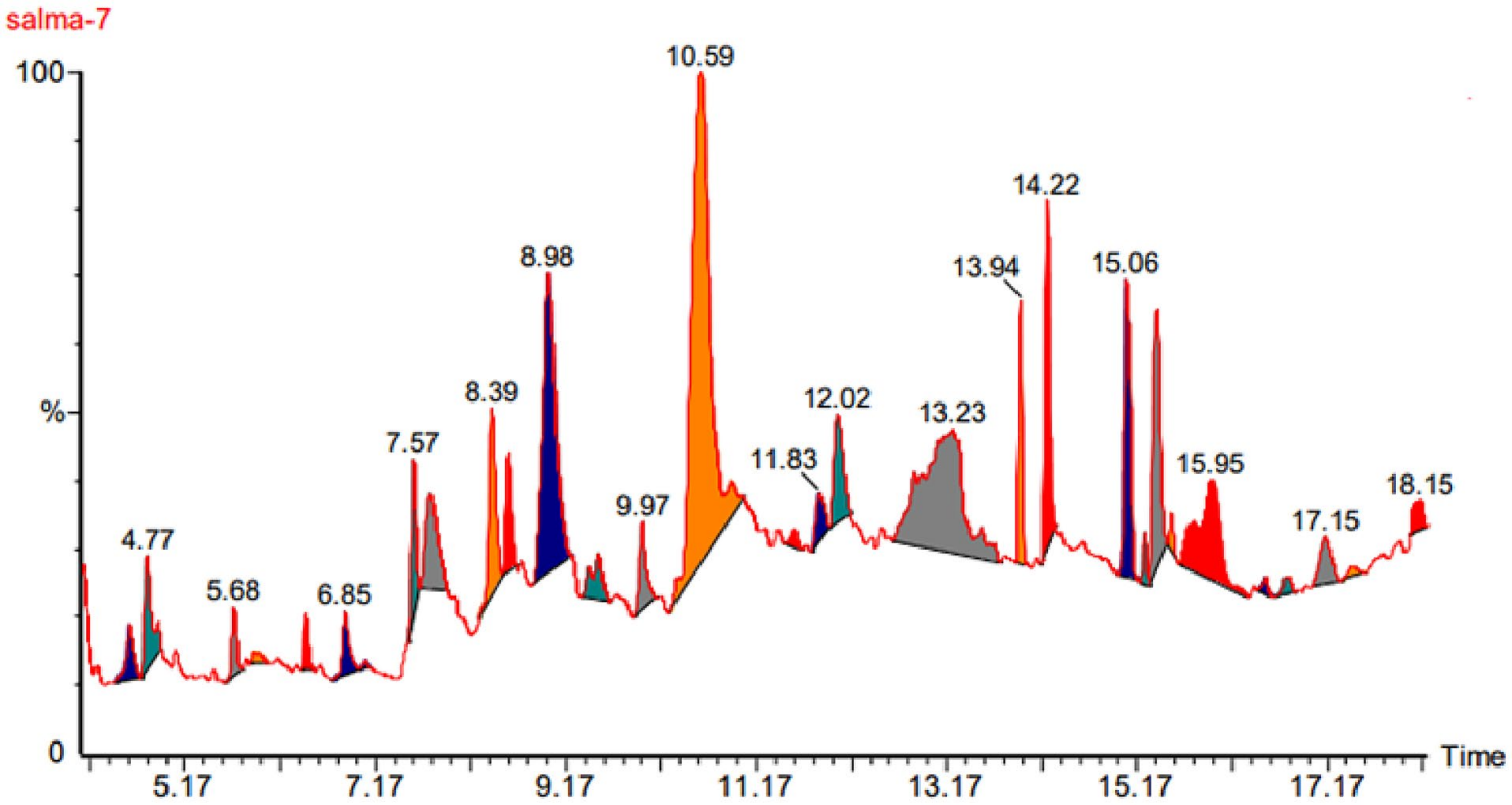
Phytochemical analysis peaks of the methanolic extract of *C. papaya* by GC–MS.

The results obtained from the GC–MS analysis of the methanolic extract of *C. papaya* fruit revealed phytochemicals (Table 2; Figure 2) with considerable amounts, such as Xanthosine and Decanoic Acid (28.190 and 14.130% respectively) of the total peak area, 3-Hydroxy-4-Methyl Pentanoic Acid, Dl-Glyceraldehyde Dimer, 2-(2-Acetoxy-1-Propyl)-2,5- Dimethoxytetrahydrofuran and 2,3-Dihydroxy-Propanal (8.840, 7.250, 7.140 and 6.550% respectively) of the total peak area, Pseduosarsasapogenin-5,20-Diene, 2,3-Dihydro-3,5-Dihydroxy-6- Methyl-4 h-Pyran-4-One, 2-Methyl Propanoic Acid, 9,12-Octadecadienoyl Chloride, Cytidine, and 2,4-Dihydroxy-2,5-Dimethyl-3(2h)-Furan-3-One (4.710, 3.530, 2.570, 2.500, 2.060, and 1.190% respectively) of the total peak area. The lowest amounts of the compounds that found in *C. papaya* extract are Cyclopropanepentanoic Acid, 18-Nonadecen-1-Ol and Dihydrotorulosol (0.810, 0.600 and 0.170% respectively).

**Table 2:** Phytochemical analysis of the methanolic extract of *C. papaya* by GC–MS.

Name	RT	Area %	Area
2-Methyl Propanoic Acid	4.59	2.570	173,835
Dl-Glyceraldehyde Dimer	4.77	7.250	489,827
2,4-Dihydroxy-2,5-Dimethyl-3(2h)-Furan-3-One	5.68	1.190	80,507
2,3-Dihydro-3,5-Dihydroxy-6- Methyl-4H-Pyran-4-ONE	7.57	3.530	238,561
2,3-Dihydroxy-Propanal	7.76	6.550	442,256
2-(2-Acetoxy-1-Propyl)-2,5- Dimethoxytetrahydrofuran	8.39	7.140	481,845
3-Hydroxy-4-Methyl Pentanoic Acid	8.56	8.840	596,660
Xanthosine	10.59	28.190	1,903,607
Cytidine	12.03	2.060	138,932
Decanoic Acid	13.29	14.130	954,340
Methyl Ester of Heptadecanoic Acid	13.94	3.540	238,908
Hexadecanoic Acid	14.22	3.280	221,463
(Z,Z)-Heptadeca-8,11-Dien-1-YL	15.06	2.300	155,173
Cyclopropanepentanoic Acid	15.25	0.810	54,494
9,12-Octadecadienoyl Chloride	15.37	2.500	168,856
Pseduosarsasapogenin-5,20-Diene	15.96	4.710	318,230
Dihydrotorulosol	17.46	0.170	11,423
18-Nonadecen-1-ol	18.09	0.600	40,688

### MTT results

**Figure 3:**
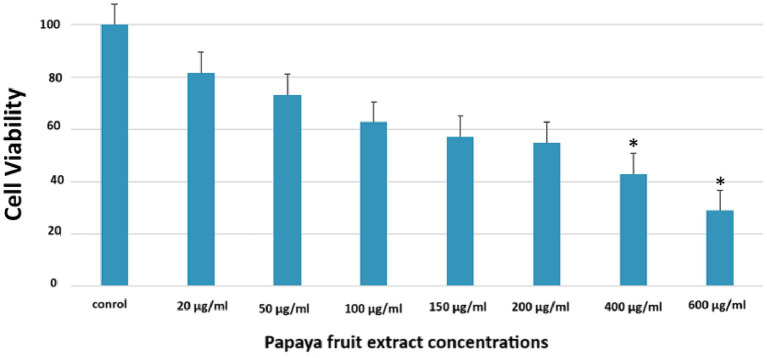
The treatment induced cytotoxicity in HUH-7 cell lines in a dose-dependent manner. There was a reduction of (MTT) (%) in 48 h.

**Figure 4:**
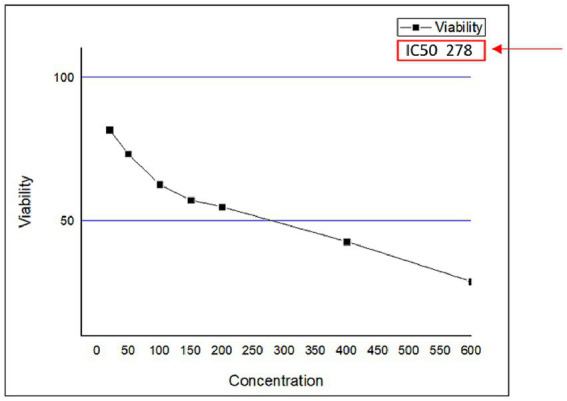
The (IC50) was determined by the dose–response curve graph using the program (Origen Pro 8.5).

If we now turn to measure the cytotoxicity of *C. papaya* fruit extract on metabolic activity, cellular viability, and proliferation. The results showed that the fruit extract exhibited lower cytotoxicity at concentrations of 20 μg/mL, 50 μg/mL, 100 μg/mL, 150 μg/mL, and 200 μg/mL (Table 3; Figure 3). In comparison to the control group of unexposed cells, all concentrations led to a reduction in cell survival. However, the most pronounced decrease in cell viability occurred at the last two concentrations: 400 and 600. At these concentrations, cell viability dropped to 42 and 30%, respectively. (Table 3; Figure 3). On the other side, significantly induced cell viability compared to the control group. Additionally, the IC50 concentration of the papaya fruit extract, which is the concentration of a drug needed to inhibit a biological process or response by 50% of the HuH-7, showed that the cells’ viability decreased significantly as the concentration of the extract increased (Figure 4). Results from the MTT assay indicated a considerable loss of membrane integrity after exposure of the cells to the tested fruit extract when compared to untreated cells after 48 h.

**Table 3:** The treatment induced cytotoxicity in HUH-7 cell lines in a dose-dependent manner.

	Contol	20 μg/mL	50 μg/mL	100 μg/mL	150 μg/mL	200 μg/mL	400 μg/mL	600 μg/mL
Viability	100.0000	81.63967	73.19204	62.65587	57.16959	54.83168	42.76809	28.86534
STD	0.20641	0.033600	0.085967	0.088425	0.053200	0.073364	0.043385	0.047606
*P* value		0.194513	0.151827	0.244431	0.547125	0.764587	0.031988	0.090027

There was a reduction of (MTT) (%) in 48 h.

### The antifungal effect of papaya’s fruit extract on *Microsporum canis*’s including ZI, MIC, MFC, and cell morphology’s images by SEM and TEM

**Figure 5:**
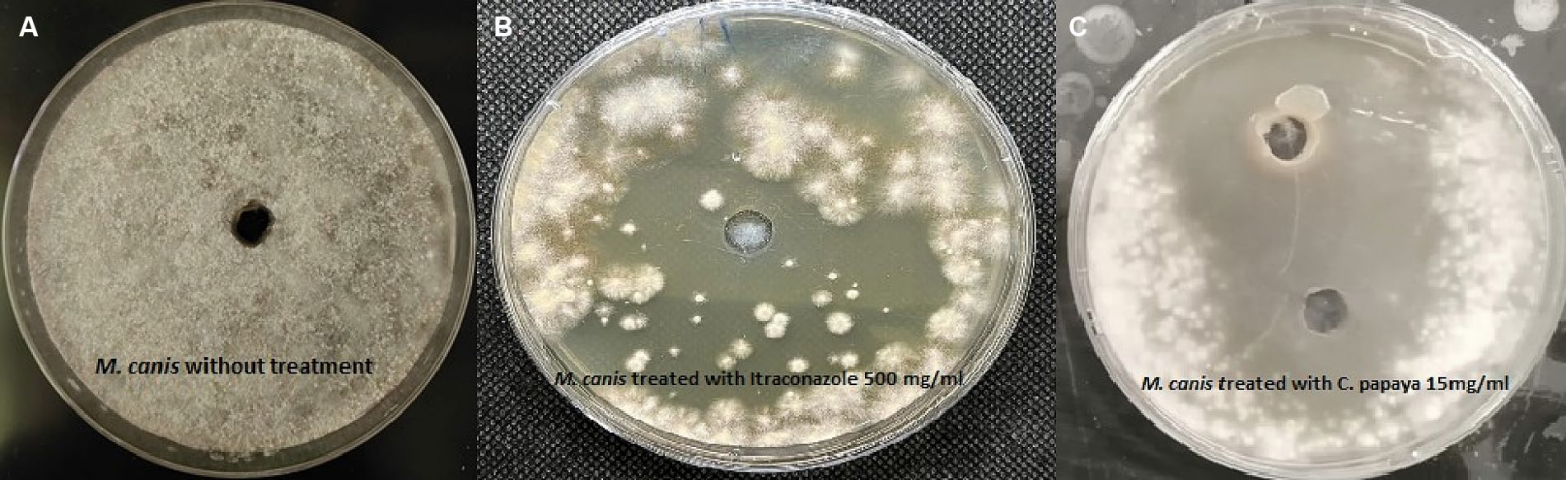
The inhibition of zone showed by *M. canis* when treated by the antibiotic and Papaya’s extract **(A)** *M. canis* without treatment, **(B)** *M. canis* treated with Itraconazole 500 mg/mL, **(C)** *M. canis* treated with *C. papaya* extract 15 mg/mL.

**Figure 6:**
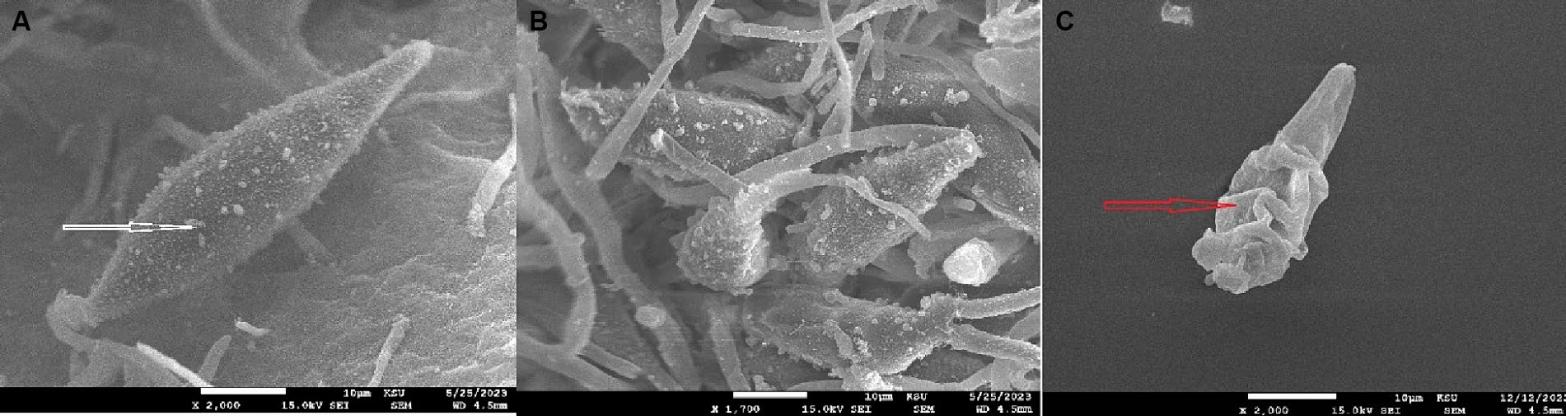
**(A)** *Microsporum canis’s* spores appear clear oval shape of the spore and the white arrow focus on the papillary nodules. **(B)** *M. canis* treated with antibiotic which appeared the similar size of spores as the normal and the papillary nodules quietly decreased. **(C)** *M. canis* treated with *C. papaya* extract which revealed significant changes in spore’s shape including: decrease in size, shrinkage, surface wrinkles and no papillary nodules appear.

**Figure 7:**
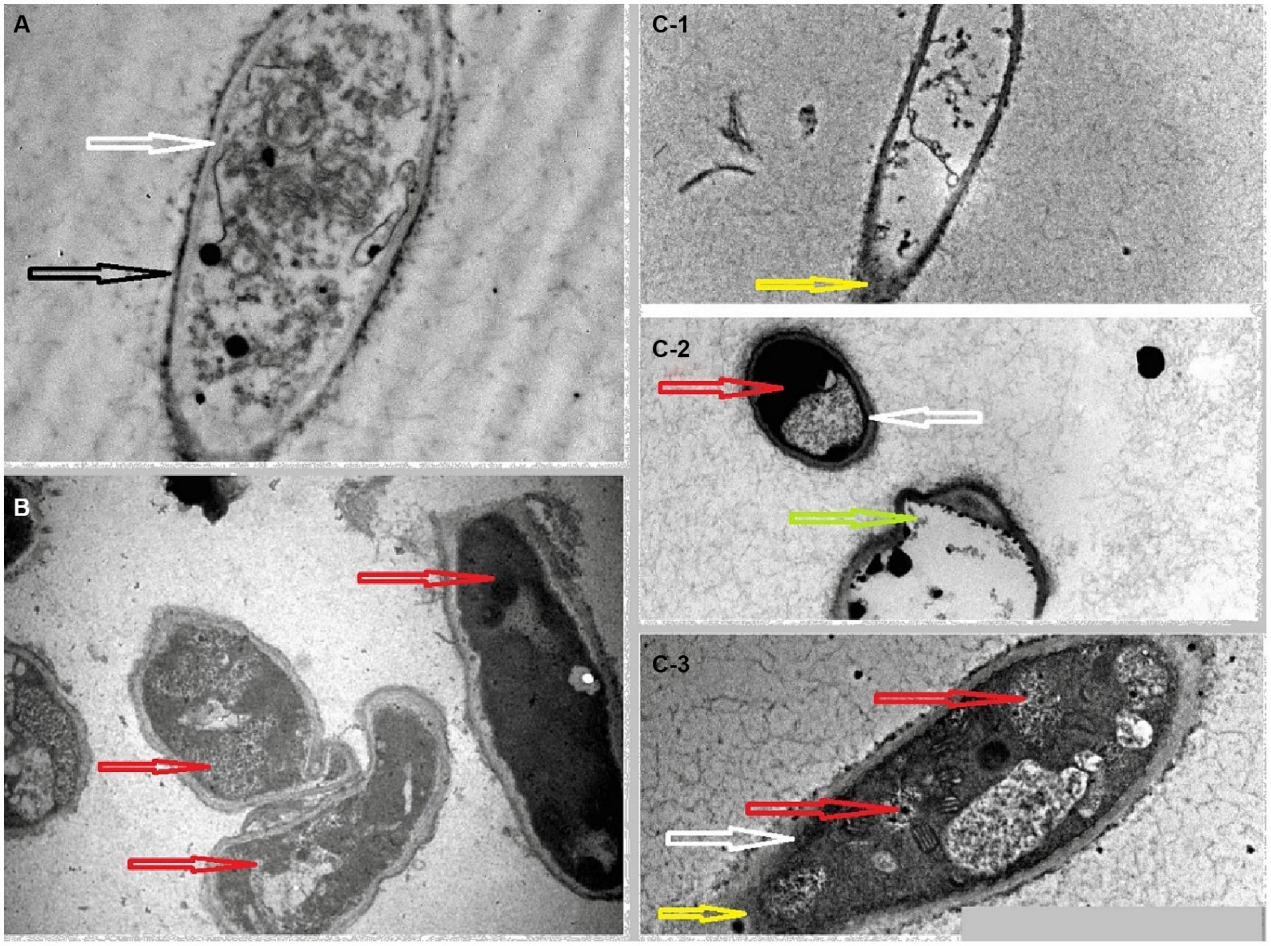
**(A)** Normal spores of *M. canis* which presented a clear cell wall (black arrow) and cell membrane (white arrow) the papillary nodules appear as black dots in the cytoplasm. **(B)** The effects on hyphae and spores of *M. canis* after the treatment with antibiotic. **(C)** *M. canis’s* spore and hyphae treated by *C. papaya* extract. In photo B and C the red arrow revealed considerable amount of lipid droplets in the cytoplasm, the green arrow showed a prominence in the spore and the yellow arrow presented rupture in the cell wall lead to escape in cytoplasm.

After conducting tests to determine the toxicity of Papaya fruit’s methanolic extract on HUH-7 cells, we found that concentrations of 400 μg/mL and 600 μg/mL were not conducive to viable cells. However, the results of the MIC test showed that 1 mg/mL or 1,000 μg/mL concentrations were effective on *M. canis’s* cells. Based on susceptibility tests that yield MIC 1 mg/mL (Table 4), phytochemicals are commonly categorized as antimicrobials (Simoes et al., 2009). The most surprising aspect of the data is in the zone of inhibition which revealed wide inhibition zone (37 mm) (Figure 5), followed by MFC which showed 1.9 mg/mL. In the initial week of the experiment, Itraconazole, which was used as a control to treat *M. canis*, demonstrated a broad zone of inhibition. However, subsequent hyphal growth within this inhibited area indicated that the fungi developed resistance to the antibiotic after just one week. We utilized SEM and TEM to observe the cellular changes in *M. canis* following treatment with papaya fruit and antibiotic. Figure 6, illustrates the significant effect of the methanolic extract from papaya fruit on the spores of *M. canis*. Image A shows a typical spore, normal in size with clear and regular papillary nodules. Image B shows the spores post-treatment with Itraconazole, which did not significantly change the *M. canis* spores. However, image C reveals significant changes in the spores following treatment with the papaya extract. These changes include a reduction in size, shrinkage, surface wrinkles, and the absence of papillary nodules, indicating a complete transformation in the shape of the spores. Following the completion of the antimicrobial experiments, we applied TEM imaging to examine the internal alterations that occurred post-treatment with the antibiotic and Papaya extract. Figure 7A demonstrates that the cell wall and cell membrane are intact, and the nuclei, vacuole, and mitochondria maintain their normal shape in the macroconidia. However, Figure 7B shows a significant presence of lipid droplets and a decline in cell size. In Figure 7C3, the cell membrane appears to have deteriorated, and the entire intracellular space is filled with lipid droplets, representing a separation of the cytoplasm from the outer cell envelope. Both images C3 and C2 display dark, electron-dense areas. Moreover, a distinct prominence is evident in the C2 image.

**Table 4:** The antifungal effect of Papaya’s fruit extract on *M. canis*’s including ZI, MIC, MFC.

	Zone of inhibition	MIC	MFC
*C. papaya* against *M. canis*	37 mm	1.0 mg/mL	1.9 mg/mL

### Effect of papaya on *Microsporum canis* infection in skin tissue

**Figure 8:**
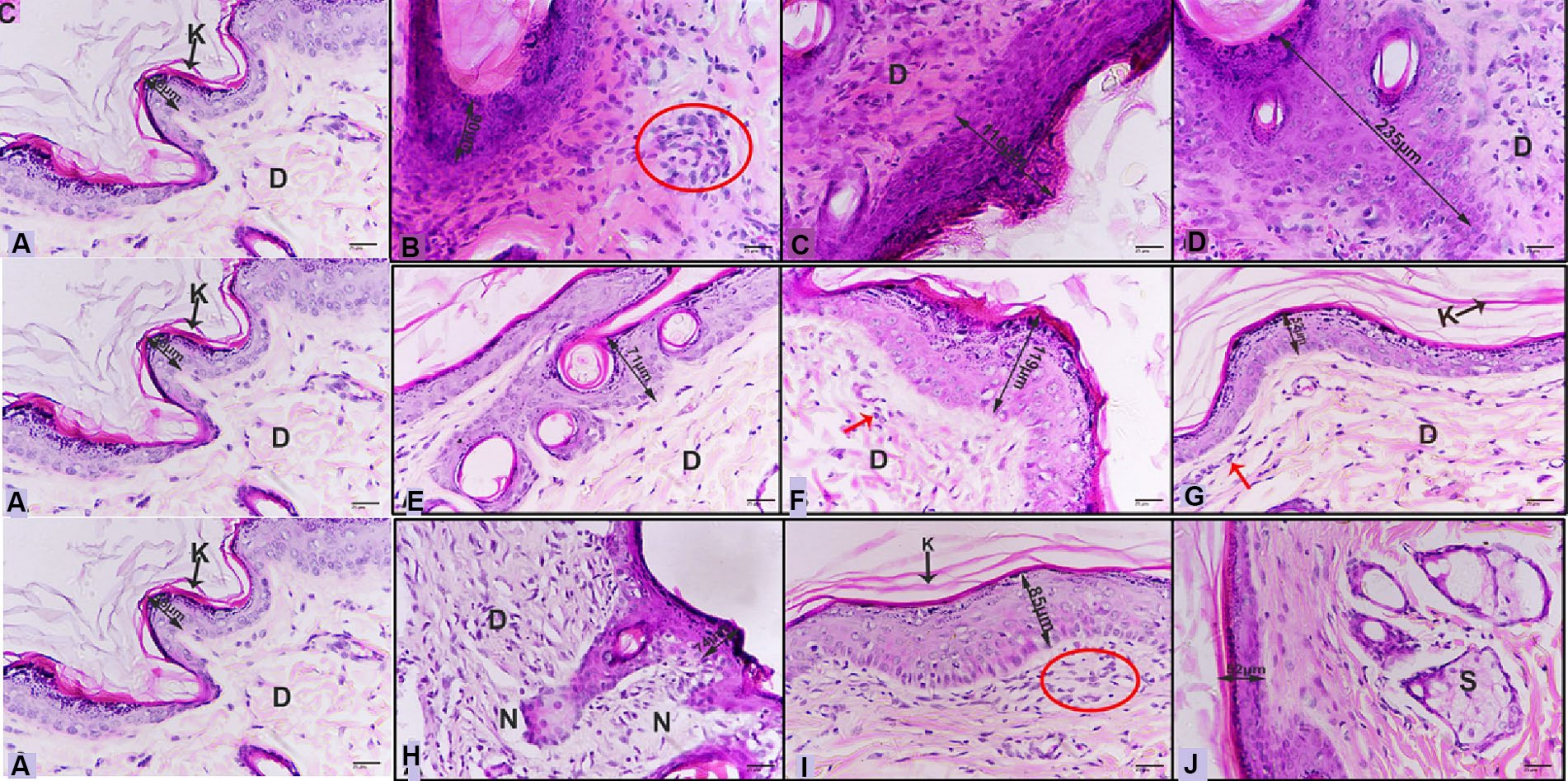
**(A)** Photomicrograph of control skin showing normal view, normal epidermis layer (double head arrow), keratinized layer (arrow and K), dermis (D). Photomicrographs of infected skin with *M. canis* **(B)** after 6 days revealed thick epidermis (double head arrow), infiltrative cells with fibroblast cells (red circle), **(C)** after 9 days displayed thicker epidermis (double head arrow), dermis layer enriched with infiltrative cells and fibroblasts (D), **(D)** after 15 days showing more thicker epidermis (double head arrow), dermis with infiltrative cells (D). **(E)** after 6 days revealing epidermis (double head arrow), dermis with less infiltrative cells (D), **(F)** after 9 days displaying thicker epidermis (double head arrow), dermis (D) layer with some infiltrative (red arrow), **(G)** after 15 days showing healthy epidermis (double head arrow), keratinized layer (arrow and K) dermis (D) with a few numbers of infiltrative cells (red arrow). **(H)** after 6 days exhibiting epidermis (double head arrow), dermis with infiltrative cells (D), necrotic foci (N), **(I)** after 9 days posting thicker epidermis (double head arrow), keratinized layer (arrow and K), dermis (D) layer with infiltrative cells with fibroblasts (red circle), **(J)** after 15 days showing healthy epidermis (double head arrow), sebaceous gland (S) (H&E-400X).

Control skin showed normal view of epidermis with thin length (Table 5), and dermis with fibroblasts and collagenous fibers (Figure 8A). Meanwhile, untreated infected skin with *M. canis* after 6 days revealed congested tissue with thick epidermis and dermis displayed aggregations of infiltrative cells (Figure 8B), after 9 days infection was stronger, epidermis was thicker and infiltrative cells were increased (Figure 8C), after 15 days epidermis was thicker, and infiltration was heavy (Figure 8D). Moreover, infected skin treated with anti-fungal drug showed marked curing improvement in all stages (Figures 8E–G). Furthermore, infected skin treated with papaya also posted curing improvement, after 6 days dermis layer showed infiltration and necrotic foci (Figure 8H), after 9 days epidermis was thicker and dermis contained marked infiltration (Figure 8I), after 15 days epidermis became normal with less infiltration (Figures 8H,J; Table 5).

**Table 5:** Epidermal length of experimental groups compared to control group after 6, 9, and 15 days.

Groups	6 days μm × 10^6^	9 days μm × 10^6^	15 days μm × 10^6^
C	48 ± 24	48 ± 24	48 ± 24
Infected without treatment	90 ± 6^*a^	116 ± 57^*a^	235 ± 16^*a^
Infected treated with anti fungi	72 ± 24^*a,b^	119 ± 57^*a^	52 ± 16^*a,b^
Infected treated with papaya extract	74 ± 28^*a,b^	85 ± 36^*a,b^	52 ± 17^*a,b^

Data = Mean ± SEM, *p* ≤ 0.05 considered sig., *a sig. Compared to control, *b sig. Compared to micro group.

## Discussion

The project initially aimed to find a natural product that could effectively combat the agent causing tinea capitis, both *in vitro* and *in vivo*. Upon reviewing existing literature, no studies were found that investigated the antifungal properties of *C. papaya* fruits. After extracting *C. papaya* fruits using methanol, we identified a significant amount of key chemical compounds that could potentially aid in treating fungal skin infections. The FTIR analysis revealed the presence of alcohol, amines, alkenes, and alkyl ether groups. The GC–MS analysis identified compounds with potential antibacterial, antioxidant, and anti-inflammatory properties. The compound with the highest percentage isolated from the papaya extract was 18-Nonadecen-1-ol (18%), which is believed to play a role in treating fungal infections. This aligns with the findings of Markovi´, Barbari´, and Brala (Karković Marković et al., 2019), who reported that 18-Nonadecen-1-ol possesses significant antimicrobial activity. Hihydrotorulosol, another compound found in considerable amounts in the papaya extract, could potentially inhibit fungal growth. This research corroborates the findings of Owolabi and others, who reported that hexadecanoic acid can reduce the production of nitric oxide, a key mediator in inflammation processes (Owolabi et al., 2018). This could explain why tissues treated with papaya ointment showed more improvement than those treated with commercial ointment. Furthermore, it was found that Hexadecanoic acid has antibacterial activity by damaging the cell walls of *S. typhi* (Johannes and Litaay, 2016). These results support our hypothesis about the antibacterial properties of Hexadecanoic acid. Papaya extract showed lower cytotoxicity at concentrations ranging from 20 μg/mL to 200 μg/mL and Higher concentrations of 400 μg/mL and 600 μg/mL showed moderate cytotoxicity. The MTT assay results indicated a significant loss of membrane integrity in cells exposed to the extract compared to untreated cells after 48 h. Looking at the antifungal properties of papaya extract, we observed a significant inhibition zone (37 mm). This finding could validate the existence of the a forementioned chemical compounds. The MIC and MFC results were presented a promised antifungal activity by papaya’s fruit, these are interesting findings which reported for the first time. Several recent studies reported that Papaya’s seeds and leaves have antimicrobial activity. Sherwani and his group, tested papaya’s leaf extract against selected 6 saprophytic fungi *Penicillium* sp.*, Aspergilus flavus*, *Aspergillus niger*, *Fusarium* sp.*, Rhizopus* and *Helminthosporum*, 5 dermatophytic fungi *Microsporum canis*, *Microsporum gypseum*, *Trichophyton rubrum*, *Trichophyton mentagrophytes*, *Trichophyton tonsurans* and 6 yeasts including *Candida albicans*, *Candida albicans* ATCC 0383, *Saccharomyces cerevisiae*, *Candida galbrata*, *Candida tropicalis*, *Candida kruzei*, Their findings revealed that the activity was effective against most fungi, however, it was significantly more potent when using the crushed leaf extract (Sherwani et al., 2013). A different research project examined the ethanolic extract of *C. papaya* and its effects on *Escherichia coli* and *Aspergillus flavus*. The study discovered that the extract exhibited significant antifungal properties when applied at a 6% concentration over a period of four days. Interestingly, the researchers observed that the ethanolic extract of *C. papaya* demonstrated stronger antifungal properties compared to its antibacterial properties. This suggests that it could be more effective in treating certain fungal infections caused by *Aspergillus flavus*, similar the effects of n-hexane extracts (Dozie-Nwakile et al., 2020). There are similarities between these findings and our results. Additionally, the methanolic extract of *C. papaya* activity against fungi supports evidence from clinical observations (Singh and Ali, 2012; Nilofer and Chenthamarai, 2021). During the first week of the experiment, Itraconazole, employed as a control for treating *M. canis*, showed a wide area of inhibition. Later, the fungi showed signs of resistance to the antibiotic as verified by the hyphal growth within the previously inhibited area, and this occurred in just a week’s time the reason for this could be that the tested organism was isolated from the hospital, which exposed it to numerous antibiotics, leading to the development of antibiotic resistance. After confirming the promising anti-dermatophyte activity of papaya fruit extract, we proceeded to investigate its impact on the hyphae and spores of *M. canis*. This study involved the use of SEM and TEM images. SEM photos shown a reduction in hyphal size, shrinkage, surface wrinkles, and the absence of papillary nodules, demonstrating a complete transformation in the shape of the spores. TEM results showed a significant presence of lipid droplets and a decline in cell size. The cell membrane appeared to have deteriorated, and the entire intracellular space was filled with lipid droplets, signifying a separation of the cytoplasm from the outer cell envelope. Additionally, dark, electron-dense areas were observed. Notably, four other studies reported similar findings regarding fungi hyphae and spores using SEM and TEM, although they used different types of plant extracts and fungi (Ghannoum et al., 2012; Aala et al., 2014; Mihalache et al., 2018; Abdallah et al., 2023). The last part of the project’s experiments is an *in vivo* result which agree with the findings of the *in vitro* results. We conducted the animal experiments by dividing them in to three groups: first, control group which effected by *M. canis* and left without any treatment. Second, effected and treated with Canesten ointment, and the last group is affected and treated with the papaya extract ointment. It was surprising that the papaya ointment treated group revealed significant improvement of *M. canis* infection from those of the control group and treated by Canesten ointment. *In vivo* results supported the *in vitro* findings. Further work is needed to identify or develop drugs that can irradicate the other dermatophytes infection.

In summary, this project investigated the antifungal properties of methanolic extracts from *C. papaya* fruits. The study revealed promising outcomes in treating tinea capitis infection both *in vitro* and in experimental animals. These findings encourage further exploration of the active compounds present in papaya fruits for potential use in treating various microbial infections.

## Data availability statement

The original contributions presented in the study are included in the article/supplementary material, further inquiries can be directed to the corresponding author.

## Ethics statement

Ethical approval was not required for the studies on humans in accordance with the local legislation and institutional requirements because only commercially available established cell lines were used. The Animal care and use committee at King Saud University granted approval for all animal experiments (Ethics Reference Number: KSU-SE-1978). The study was conducted in accordance with the local legislation and institutional requirements.

## Author contributions

SalA: Investigation, Methodology, Writing – review & editing. HR: Conceptualization, Writing – review & editing. AA: Investigation, Writing – review & editing. SaA: Methodology, Writing – review & editing. GA: Methodology, Writing – review & editing. HA: Methodology, Writing – review & editing. RE: Data curation, Investigation, Methodology, Writing – original draft.
